# AI-Assisted Fusion Technique for Orthodontic Diagnosis Between Cone-Beam Computed Tomography and Face Scan Data

**DOI:** 10.3390/bioengineering12090975

**Published:** 2025-09-14

**Authors:** Than Trong Khanh Dat, Jang-Hoon Ahn, Hyunkyo Lim, Jonghun Yoon

**Affiliations:** 1 Faculty of Mechanical Engineering, Ho Chi Minh City University of Technology (HCMUT), 268 Ly Thuong Kiet, Dien Hong Ward, Ho Chi Minh City 700000, Vietnam; ttkdat@hcmut.edu.vn; 2 Vietnam National University Ho Chi Minh City (VNUHCM), Linh Xuan Ward, Ho Chi Minh City 700000, Vietnam; 3 Department of Orthodontics, Chungang University Gwangmyeong Hospital, Gwangmyeong 14353, Gyeonggi-do, Republic of Korea; ajh0225@gmail.com; 4 Department of Mechanical Engineering, Hanyang University, 55, Hanyangdaehak-ro, Sangnok-gu, Ansan-si 15588, Gyeonggi-do, Republic of Korea; dlaodry9@gmail.com; 5 BK21 FOUR ERICA-ACE Center, Hanyang University, Ansan-si 15588, Gyeonggi-do, Republic of Korea

**Keywords:** pose normalization, point-to-plane registration, multimodal image fusion, surface-to-volume alignment, craniofacial modeling

## Abstract

This study presents a deep learning-based approach that integrates cone-beam computed tomography (CBCT) with facial scan data, aiming to enhance diagnostic accuracy and treatment planning in medical imaging, particularly in cosmetic surgery and orthodontics. The method combines facial mesh detection with the iterative closest point (ICP) algorithm to address common challenges such as differences in data acquisition times and extraneous details in facial scans. By leveraging a deep learning model, the system achieves more precise facial mesh detection, thereby enabling highly accurate initial alignment. Experimental results demonstrate average registration errors of approximately 0.3 mm (inlier RMSE), even when CBCT and facial scans are acquired independently. These results should be regarded as preliminary, representing a feasibility study rather than conclusive evidence of clinical accuracy. Nevertheless, the approach demonstrates consistent performance across different scan orientations, suggesting potential for future clinical application. Furthermore, the deep learning framework effectively handles diverse and complex facial geometries, thereby improving the reliability of the alignment process. This integration not only enhances the precision of 3D facial recognition but also improves the efficiency of clinical workflows. Future developments will aim to reduce processing time and enable simultaneous data capture to further improve accuracy and operational efficiency. Overall, this approach provides a powerful tool for practitioners, contributing to improved diagnostic outcomes and optimized treatment strategies in medical imaging.

## 1. Introduction

In contemporary society, driven by rapid advancements in medical expertise and computer technology, there has been a notable increase in the use of diverse medical imaging modalities. These include a wide range of techniques, such as computed tomography (CT), magnetic resonance imaging (MRI), and facial scanning, all aimed at enhancing diagnostic accuracy and visualizing anticipated treatment outcomes. Differences in acquisition equipment and imaging methodologies lead to variations in initial poses and clinical interpretations across multimodal medical images. Spiral CT is distinguished by its precision in reconstructing bone tissue using distinct X-ray absorption coefficients, offering exceptional accuracy in bone reconstruction. Conversely, although MRI provides high accuracy for soft tissue reconstruction, its drawbacks include lengthy acquisition times and relatively high costs. In contrast, facial scanning offers the advantage of capturing intricate details, such as high-resolution color and texture features, thereby augmenting diagnostic capabilities in unique ways.

In cosmetic surgery and orthodontics, the integration of cone-beam computed tomography (CBCT) with facial scanning data represents a groundbreaking advancement with multiple benefits. By combining CBCT, which provides detailed insights into dental and skeletal structures, with facial scanning, which captures intricate facial features and soft tissue dynamics, practitioners can obtain a comprehensive understanding of patients’ anatomical characteristics. This holistic approach facilitates precise treatment planning and execution, resulting in improved surgical outcomes and increased patient satisfaction. One of the primary advantages of fusing CBCT and facial scanning data is its ability to provide three-dimensional visualization of the craniofacial complex. This comprehensive view enables surgeons and orthodontists to accurately assess the interplay between facial aesthetics and underlying skeletal structures, facilitating personalized treatment strategies tailored to each patient’s unique anatomy. Moreover, the fusion of CBCT and facial scanning data streamlines the diagnostic process by providing a seamless workflow from preoperative planning to postoperative evaluation. Clinicians can precisely simulate surgical interventions and orthodontic movements in virtual environments, enabling meticulous preoperative analysis and optimization of treatment plans. Subsequently, during surgical or orthodontic procedures, this preplanned approach translates into greater efficiency and accuracy, minimizing intraoperative complications and optimizing surgical outcomes.

The integration of multimodal medical images has shown significant utility in various medical applications. For example, Monfardini et al. [1] applied ultrasound and cone-beam CT fusion techniques for liver ablation procedures, while Sakthivel et al. [2] used PET-MR fusion for postoperative surveillance. Furthermore, Dong et al. [3] demonstrated the effectiveness of CT-MR image fusion in reconstructing complex 3D extremity tumor regions. Kraeima et al. [4] incorporated multimodal image fusion into a workflow for 3D virtual surgical planning (VSP), highlighting its potential to enhance surgical precision. However, it is worth noting that although CT visualization is currently the standard in routine 3D VSP procedures [5], the inclusion of additional modalities, such as MRI, holds promise for further refining surgical planning and execution.

A neural network for landmark detection introduces a feature point extraction module, which is employed within the algorithm to explain the process of learning feature extraction and to facilitate subsequent registration tasks. A fundamental distinction between processing 3D point cloud data and traditional 2D planar image data is the inherent disorder of point cloud data, in contrast to the sequential relationships used in image convolution operations. In addition, 3D point cloud data exhibit spatial correlation and translational–rotational invariance, which present challenges for keypoint extraction. Traditional methods for 3D point cloud feature extraction often involve dividing the data into voxel grids with spatial dependence [6] or using recurrent neural networks to eliminate input point cloud data sequences [7]. However, these approaches may result in unnecessarily large data representations and the associated computational burden. PointNet [8] introduced a novel neural network architecture designed to process point cloud data directly while preserving the permutation invariance of the input points. This enables effective classification and semantic segmentation of scenes without the need for voxelization or image conversion. However, PointNet has limitations in capturing the local features of point cloud data, which hinders its ability to effectively analyze complex scenes and limits its generalization capabilities.

The Iterative Closest Point (ICP) algorithm is a widely used method for point cloud registration, particularly for rigid model transformations. At its core, the algorithm operates by greedily establishing correspondences between two point clouds and then applying the least-squares method to determine the parameters for rigid transformation. Although originally designed for rigid registration, the ICP algorithm has been extended to accommodate nonrigid transformations [9], in which adjustable ground rigidity parameters are introduced to optimize the transformation under specified rigidity constraints. In practice, optimization of the ICP algorithm often involves approximate least-squares methods, which can be computationally intensive [10]. Despite its effectiveness, ICP presents challenges, notably its susceptibility to local minima and dependence on initialization variables. Various strategies have been proposed to mitigate these issues. For example, Go-ICP [11] employs a branch-and-bound approach to explore the entire 3D motion space, achieving global optimization by bounding a new error function. However, this method incurs significant computational overhead compared to traditional ICP, even with local acceleration.

Nevertheless, several challenges arise due to the disparities between CBCT and facial scanning data, which affect the fusion process. First, during acquisition, facial scanning captures information related to hair and clothing, which is absent in CBCT data. Second, while CBCT includes both soft tissue and bone density information, facial scanning primarily provides surface details and color information. Third, because CBCT and facial scanning data are not synchronized, variations in patients’ facial expressions and gestures between the two datasets are inevitable.

To address these challenges, we propose a novel methodology that effectively fuses disparate data types while ensuring precision and coherence in the integration process. Unlike conventional approaches that typically focus on detecting specific features and aligning corresponding points between datasets, our method leverages a pre-trained deep learning model (MediaPipe) without additional re-training. This model facilitates the detection of facial meshes in both CBCT and facial scanning data and subsequently aligns their positions and orientations according to a predefined standard. An iterative closest point (ICP)–based algorithm is then employed to refine the registration of the transformed data and ensure accurate fusion. Finally, the results were validated based on the achieved accuracy, which served as the benchmark for evaluation and quality assessment. Beyond technical accuracy, multimodal fusion of craniofacial data raises important questions of data privacy and governance. Recent work has highlighted the ethical and cybersecurity risks of handling identifiable facial data and emphasized the need for privacy-by-design strategies when developing AI-based registration pipelines [12]. In this study, we address registration feasibility while also acknowledging the importance of integrating robust consent procedures and ethical safeguards in future clinical deployments.

## 2. Method

### 2.1. Data Preparation

The data preparation phase involved the collection of CBCT and facial scan data from three male participants (ages 24–34 With regard to the data acquisition process for the three participants in this study, we clarify that the data were obtained without issues in accordance with the standard medical imaging procedures routinely performed at the accredited Chung-Ang University Hospital. All three subjects were used consistently for both qualitative demonstration and quantitative evaluation. Each dataset contained 3D scanning data consisting of 576 slices at a resolution of 768 × 768 × 576 pixels, with a slice thickness of 0.3 mm. Full field-of-view (230 × 170 mm) images were acquired using an I-CAT CBCT scanner (KaVo Dental GmbH, Biberach, Germany) under the following operational parameters: 120 kV, 37.1 mA, voxel size of 0.3 mm, and a scan time of 8.9 s. Face scans were obtained using a Zivid-2 structured-light RGB-D camera (Zivid Co., Ltd., Oslo, Norway), which operates on the structured-light principle and produces RGB-D data (RGB image + depth). Acquisition parameters: distance ~0.8 m, exposure 6 ms, resolution 1920 × 1200, triangulation accuracy ~0.2 mm. The CBCT and facial scans were independently collected, not simultaneously, to ensure clarity in the acquisition protocol. This combination of high-resolution CBCT and 3D facial scanning provided a comprehensive dataset for analyzing facial structures in three dimensions.

To maintain the integrity of the dataset, strict data handling protocols were implemented. Each scan underwent a quality assurance process to ensure clarity and accuracy, with any data showing artifacts or inconsistencies being reacquired. CBCT and facial scans were then anonymized and catalogued in a secure database in compliance with data protection regulations. To prepare the data for the feature point learning algorithms, the raw scans were processed to align the CBCT and facial scan data, normalize the scales, and convert the data into a consistent format for subsequent analyses. This meticulous preparation was crucial for the success of the feature extraction and machine learning phases, ensuring that the dataset was robust, reliable, and ready for complex analytical tasks.

Participant demographics: all three were male, aged 24–34. Inclusion criteria: no prior facial surgery or orthodontic appliances. Exclusion criteria: significant facial hair or medical conditions altering craniofacial anatomy. Pre-scan instructions included neutral facial expression, removal of glasses, and hair tied back to avoid occlusion of landmarks. These details are summarized in Supplementary Table S1.

### 2.2. Overall Flowchart for Automatic Registration

**Figure 1:**
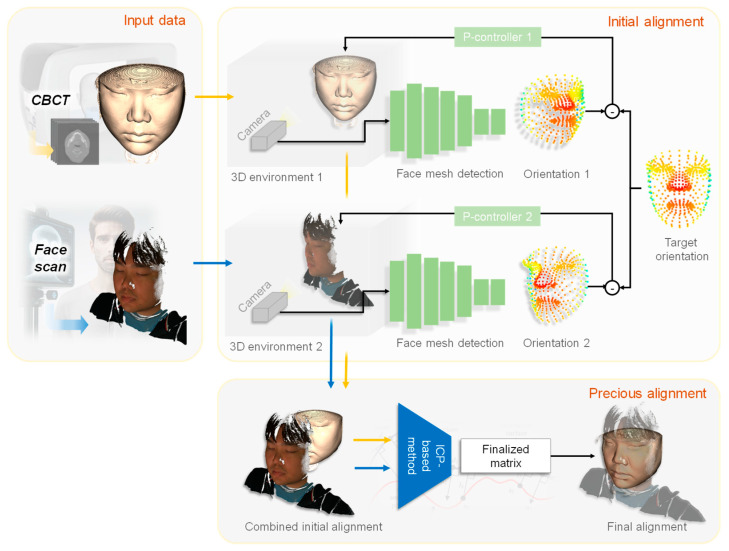
Overall flowchart of the developed method for CBCT-Face scan registration.

Figure 1 presents a detailed workflow designed for facial recognition and alignment, utilizing two distinct 3D environments derived from CBCT and facial scans. These environments are constructed from point cloud data obtained from the CBCT and facial scanners. Each environment incorporates a virtual camera responsible for capturing 2D images from a predefined position and orientation.

From the 2D images captured by the virtual cameras in these environments, MediaPipe [13], a widely recognized face mesh detection framework, was employed to predict the 3D feature points of the facial mesh. This prediction provides the orientation and position of the face, which serve as the reference points for matching the face to the virtual camera’s view. Subsequently, a refinement process was implemented using the ICP method to enhance registration precision. This step further optimized the accuracy of the alignment procedure.

### 2.3. Initial Alignment

After loading each input datum, including the CBCT and facial scan, into the corresponding 3D virtual environments, a camera was initially positioned at the origin of the coordinate system with its viewing direction aligned along the positive z–axis. The objective was to transform the point cloud such that its center aligned with a specific point along the z–axis, specifically at z = d, where d = 300 mm represents the distance from the camera to the center of the point cloud.

The transformation involved two main steps: calculating the centroid of the point cloud and translating the point cloud so that its centroid was positioned at coordinates (0, 0, d). The point cloud comprised n points {P_1_, P_2_, …, P_n_}, where each point P_i_ = (x_i_, y_i_, z_i_). The centroid C = (C_x_, C_γ_, C_z) of the point cloud was the average of all the points and was computed as follows:

(1) $$\begin{array}{c} C_{x}=\frac{1}{n}\sum_{i=1}^{n}x_{i},C_{y}=\frac{1}{n}\sum_{i=1}^{n}y_{i},C_{z}=\frac{1}{n}\sum_{i=1}^{n}z_{i} \end{array}$$

To align the centroid of the point cloud with the point (0, 0, d) on the z–axis, each point in the point cloud must be translated. The translation vector $\vec{T}$ is calculated by subtracting the centroid coordinates from (0, 0, d) as follows:

(2) $$\begin{array}{c} \vec{T}=\left(0-C_{x},0-C_{y},0-C_{z}\right) \end{array}$$

By applying this translation to each point, the new coordinates of each point after translation are given by:

(3) $$\begin{array}{c} P_{i}^{\prime }=\left(x_{i}+{\vec{T}}_{x},y_{i}+{\vec{T}}_{y},z_{i}+{\vec{T}}_{z}\right) \end{array}$$

In this method, a pre-trained MediaPipe model is employed to extract feature points from the face, which are then used to determine the orientation of a person in three-dimensional space. Specifically, the rotations around the x–, y–, and z–axes are calculated. In addition, the distance between two critical facial feature points, such as the top-left and top-right points, is measured to estimate the scale or size of the face in the image.

In particular, the following parameters are computed:

1. The roll (rotation around the z–axis) is calculated by analyzing the slope of the line connecting the feature points at the outer corners of the eyes, specifically landmarks 162 and 389, as shown in Equation (4).

(4) $$\begin{array}{c} \theta_{z}\left(roll\right)=\arctan\left(\frac{y_{389}-y_{162}}{x_{389}-x_{162}}\right) \end{array}$$

where ($x_{162},$ $y_{162}$) and ($x_{389},$ $y_{389}$) are the coordinates of the respective points.

2. The yaw (rotation around the y–axis) and pitch (rotation around the x–axis) are estimated using an arrangement of points on the left and right sides of the facial mesh, including landmarks 162, 234, and 132 on the left side, landmarks 389, 454, and 361 on the right side, and landmark 19 at the top of the nose, as follows:

$$\bar{x}=\frac{x_{162}+x_{234}+x_{132}+x_{389}+x_{454}+x_{361}}{6};\bar{y}=\frac{y_{162}+y_{234}+y_{132}+y_{389}+y_{454}+y_{361}}{6},$$

(5) $$\begin{array}{c} \theta_{y}\left(yaw\right)=\arctan\left(\frac{x_{19}-\bar{x}}{z_{19}}\right);\theta_{x}\left(pitch\right)=\arctan\left(\frac{y_{19}-\bar{y}}{z_{19}}\right) \end{array}$$

Distance between landmarks 234 and 454: These points are typically located on the left and right sides of the face near the outer edges of the eyes, respectively. The distance between these points serves as a reliable proxy for facial width, as follows:

(6) $$D=\sqrt{{\left(x_{454}-x_{234}\right)}^{2}+{\left(y_{454}-y_{234}\right)}^{2}+{\left(z_{454}-z_{234}\right)}^{2}}$$

The current roll, pitch, yaw, x, y, and size values of the point cloud or face are first compared with the predetermined target values. The differences between these values are then used to compute the corrections required to accurately align the point clouds with the targets. These corrections were calculated using a proportional controller (P-controller), which scales the errors by a proportional constant to determine the necessary adjustments.

Let:

– $\theta_{z}$ represent roll, $\theta_{y}$ represent yaw, and $\theta_{x}$ represent pitch.
  where D represents the current size of the face in an image.
– x,y represent the current positions of the top of the nose in the image.

The target values are:

– $\theta_{z,0}$, $\theta_{y,0}$, $\theta_{x,0}$ for orientation.
– $x_{0}$, $y_{0}$, and $D_{0}$ for position and size.

After the errors (Δ) between current and target values are computed, using a P-controller with a gain of p = 0.01. A sensitivity analysis with values ranging from 0.005 to 0.05 confirmed that this choice achieved the most stable convergence with the best trade-off between accuracy and speed (see Supplementary Table S2). The corrections are calculated as follows:

$$\delta \theta_{z}=-p\times \Delta \theta_{z};\;\;\;\;\;\;\;\Delta \theta_{z}=\theta_{z}-\theta_{z,0}$$

$$\delta \theta_{y}=-p\times \Delta \theta_{y};\;\;\;\;\;\;\;\Delta \theta_{y}=\theta_{y}-\theta_{y,0}$$

$$\delta \theta_{x}=-p\times \Delta \theta_{x};\;\;\;\;\;\;\;\Delta \theta_{x}=\theta_{x}-\theta_{x,0}$$

$$\delta x=-p\times \Delta x;\;\;\;\;\;\;\;\Delta x=x-x_{0}$$

$$\delta y=-p\times \Delta y;\;\;\;\;\;\;\;\Delta y=y-y_{0}$$

(7) $$\begin{array}{c} \delta z=-p\times \Delta ;\;\;\;\;\;\;\;\Delta D=D-D_{0}\; \end{array}$$

The transformation of a point cloud typically involves multiple rotations and translations, as expressed in the following equations. These transformations are often combined into a single matrix operation that can be efficiently applied to each point in the cloud, as shown in Algorithm 1.

$$R_{x}\left(\theta_{x}\right)=\left[\begin{array}{ccc} 1 & 0 & 0\\ 0 & cos(\theta_{x}) & -sin(\theta_{x})\\ 0 & sin(\theta_{x}) & cos(\theta_{x}) \end{array}\right]$$

$$R_{y}\left(\theta_{y}\right)=\left[\begin{array}{ccc} cos(\theta_{y}) & 0 & sin(\theta_{y})\\ 0 & 1 & 0\\ -sin(\theta_{y}) & 0 & cos(\theta_{y}) \end{array}\right]$$

$$R_{z}\left(\theta_{z}\right)=\left[\begin{array}{ccc} cos(\theta_{z}) & -sin\left(\theta_{z}\right) & 0\\ sin\left(\theta_{z}\right) & cos\left(\theta_{z}\right) & 0\\ 0 & 0 & 1 \end{array}\right]$$

(8) $$\begin{array}{c} T=\left[\begin{array}{c} \delta x\\ \delta y\\ \delta z \end{array}\right]\; \end{array}$$

To simplify the application of multiple transformations, they were combined into a 4 × 4 transformation matrix using homogeneous coordinates, which allowed rotation and translation to be handled in a single operation.

(9) $$M=\left[\begin{array}{cccc} & R & & T\\ 0 & 0 & 0 & 1 \end{array}\right]$$

where R is the product of the individual rotation matrices:

(10) $$R=R_{z}\left(\theta_{z}\right)R_{y}\left(\theta_{y}\right)R_{x}\left(\theta_{x}\right)$$

Transformation matrix M can be applied to each point $\vec{p}$ in the point cloud. If $\vec{p}$ is a point with coordinates (x,y,z) represented in homogeneous coordinates as ${\left[x,y,z,1\right]}^{T}$, the transformed point ${\vec{p}}^{\prime }$ is obtained by:

(11) $${\vec{p}}^{\prime }=M\cdot \vec{p}$$

**Figure 2:**
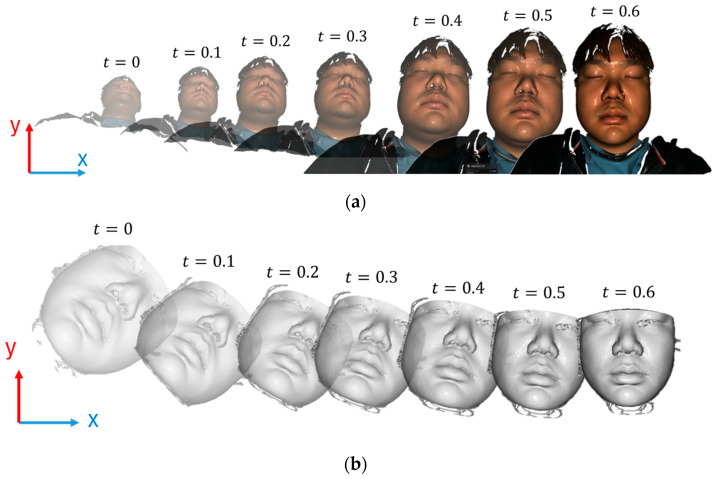
Demonstration for initial alignment process during time points: (**a**) for face scan data; (**b**) for CBCT data.

MediaPipe directly provides 3D landmark coordinates (x, y, z) defined with respect to the virtual camera system used to generate 2D projections of both CBCT and face scan data. Because both modalities are projected through the same virtual camera, their extracted 3D landmarks are inherently expressed in the same coordinate system. This enables us to use MediaPipe outputs directly for rigid pose initialization prior to ICP refinement, without any intermediate 2D-to-3D conversion. Since Figure 1 and Figure 2 already illustrate the projection and alignment workflow, no new figure was added.

This operation, summarised in Algorithm 1, is performed for every point in the cloud, effectively and efficiently realigning the entire point cloud as required, as shown in Figure 2.
**Algorithm 1**: Pseudo code for initial alignment CBCT and face scan data by face mesh detection3D initial alignment with Mediapipe1:**input**: P2:${\;\;\;\theta }_{z,0}$$,\;\theta_{y,0}$$,\;\theta_{x,0}$3:${\;\;\;x}_{0}$, $y_{0}$, and $D_{0}$4:   i=05:${\;\;\;thresh}_{1},{thresh}_{2}$6:   **repeat**
7:     i=i+18:     Detect the feature points by MediaPipe9:     Calculate $\Delta \theta_{x},\Delta \theta_{y},\Delta \theta_{z},\Delta x,\Delta y,\Delta D$
10:     Calculate $\delta \theta_{x},\delta \theta_{y},\delta \theta_{z},\delta x,\delta y,\delta D$
11:     Calculate $R_{x}\left(\theta_{x}\right),R_{y}\left(\theta_{y}\right),R_{z}\left(\theta_{z}\right),\;T$
12:     Calculate transform matrix M
13:     Update P=M·P
14:     **until** $Sum\left(\Delta \theta_{x},\Delta \theta_{y},\Delta \theta_{z}\right)<{thresh}_{1}$, 15:$\;\;\;\;\;Sum\left(\Delta x,\Delta y,\Delta D\right)<{thresh}_{2}$16:**return** P

### 2.4. Refinement via ICP Algorithm

After rough alignment. ICP (Iterative Closest Point)—a well-known local alignment method was applied for refinement.

To explain about ICP, let Q denote the source point cloud and P denote the target point cloud. For each point in Q, the algorithm attempts to find the nearest neighbour in P. If the Euclidean distance between them is less than a specified threshold, they are considered a corresponding pair and included in the set K={(p,q)}. The estimation is then proceeds to find the transformation matrix T that can be applied to the source point cloud Q such that the alignment between these two-point clouds minimizes an objective function, denoted as E(T), thereby improving the registration.

There are two primary types of ICP algorithms that use different objective functions. The first is point-to-point ICP [14], which employs the followingobjective function:

(12) $$E\left(T\right)=\sum_{\left(p,q\right)\in K}\|p-Tq{\|}^{2}$$

The second is the point-to-plane ICP [15] algorithm, which uses the objective function

(13) $$E\left(T\right)=\sum_{\left(p,q\right)\in K}(\left(p-Tq\right)\cdot n_{p}{)}^{2},$$

where $n_{p}$ is the normal of the corresponding point p. After estimating the transformation matrix T to minimize the objective function E(T), the algorithm iterates, updating the new corresponding point pairs set K. This new set includes points from the point clouds P and Q with Euclidean distances less than the threshold value. The updated set is then used to estimate another transformation T, which is applied to the source point cloud Q to minimize E(T). The algorithm repeats until the change in the objective function E(T) is less than a specified thresholdor the maximum number of iterations is reached. The final transformation matrix is the product of the transformation matrices computed over all iterations.

(14) $$T\left(threshold\right)=\prod_{n}^{i=1}T_{i}$$

where i is the number of iterations performed to meet the objective function.

Obviously, the transformation matrix depends on the threshold value set, as the threshold serves as a criterion to filter out corresponding points, and the set of corresponding points K provides the information needed to estimate the transformation matrix T. It can therefore be inferred that if the threshold value is set too high, points that do not truly correspond will be accepted, leading to a misleading estimate of the transformation matrix T. Conversely, if the threshold is set too low, the criteria become too strict and fewer points are accepted, which will similarly lead to misleading results.

**Figure 3:**
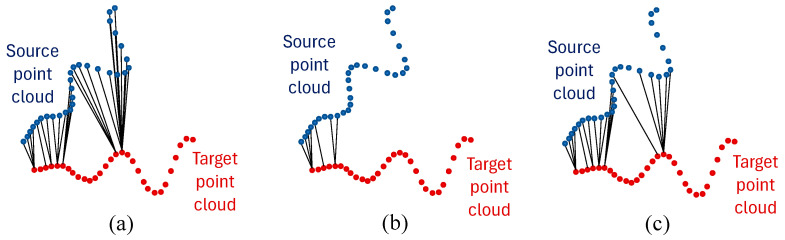
Point pairs are filtered with specific threshold values: (**a**) Too high threshold value; (**b**) Too small threshold value; (**c**) Optimal threshold.

Figure 3 clearly shows the dependence of the estimated result on the threshold value. In case (a), the threshold is too large, resulting in all points being selected and many incorrect predictions being accepted. Conversely, case (b) shows that the threshold is too small, leading to an insufficient number of points for accurately estimating the transformation matrix T. The optimal threshold value (c) lies somewhere within this range, producing a point set filter suitable for estimation. Choosing a reasonable threshold depends heavily on the input data, as there is no universal standard. Therefore, the method for determining an appropriate threshold value will be discussed in the following sections.

Owing to the particular manner in which the ICP algorithm works, it requires a sufficiently good initial alignment. Once the corresponding point-pair set K is predicted incorrectly, the estimation of the transformation matrix T will consequently be wrong, and subsequent iterations will rely on the erroneous information from the previous iteration to continue estimating. This leads to the objective function E(T) being stuck in a local minimum and unable to converge to the global minimum. Based on experiments, we found that our initial alignment step was effective in keeping the ICP algorithm on the right track.

Several studies have shown that the point-to-plane ICP algorithm offers the potential for faster convergence than traditional point-to-point ICP [16]. Therefore, in this study, we used the point-to-plane ICP algorithm implemented in the open-source library Open3D [17]. In addition, iteration consumes substantial computational resources and considerable time, therefore, we limited the iteration to a maximum of 30 cycles when the objective function E(T) had not yet reached the threshold value or when the change in E(T) was less than $10^{-6}$. his limitation reduced the time required to estimate the final transformation matrix. The results show that it reliably reduces computation time in cases without a well-aligned initialization.

### 2.5. Refinement Optimization

Based on our experimental results, a discrepancy between theoretical expectations and practical outcomes was observed. Accordingly, setting a smaller threshold value for the objective function E(T) does not necessarily lead to better registration results. Since the final goal is to assist doctors in medical diagnosis, we aim for results where the alignment is visually acceptable to the naked eye rather than merely achieving an objective function with a sufficiently small value. Therefore, a metric that can be used to evaluate the quality of alignment and automatically determine the optimal threshold value is required.

Two basic metrics were used to measure the quality of alignment between two point clouds [16,17]: (1) fitness, which represents the proportion of inlier correspondences relative to the total number of points in the source cloud (i.e., the overlap ratio between the two point clouds), and (2) inlier root mean square error (inlier RMSE), which represents the root mean square distance of all inlier pairs that satisfy the proximity condition (≤0.5 mm in this study). It should be noted that in multimodal fusion between CBCT (covering the full head volume) and a partial facial surface scan, the effective overlap region is inherently small, therefore, fitness values are expected to be lower than in unimodal or full-surface registration.

Specifically, the metrics operate after refinement with a certain threshold value has been completed, and the algorithm continues to find the corresponding point set K = {(p, q)}, just as in the previous ICP algorithm. Corresponding point pairs with a Euclidean distance of less than a set value (in this study, the set value was chosen as 0.5 mm) were considered inlier correspondences. The two evaluation parameters were calculated as follows:

(15) $$fitness\left(P,Q\right)=\frac{\mathrm{number}\;\mathrm{of}\;\mathrm{inlier}\;\mathrm{correspondences}}{\mathrm{number}\;\mathrm{of}\;\mathrm{points}\;\mathrm{in}\;\mathrm{source}\;\mathrm{cloud}}$$

(16) $$inlierRMSE\left(P,Q\right)=\sqrt{\mathrm{mean}\left(\sum {\left(p-T.q\right)}^{2}\right)}$$

where all point pairs with p−T·q less than 0.5 are included in the calculation.

To ensure efficiency, the algorithm limits the distance between corresponding pairs of points to less than a specified value. This approach minimizes the risk of falsely identifying noncorresponding points. Pairs of points that satisfy this criterion are considered inliers, indicating the true correspondence between the two point clouds. These inlier pairs were the only ones included in the evaluation parameters.

### 2.6. Reduction in Computing Time

In all the datasets used, the optimal threshold value was consistently achieved at approximately 3.0. However, to ensure that no potential threshold values were overlooked, the search range was expanded. In this study, a threshold value ranging from 0.5 to 10.0, with increments of 0.5, was chosen. Voxel downsampling with step sizes ranging from 0.5 to 1.5 mm was used adaptively to achieve stable alignment across subjects. This parameter was adjusted depending on the density of the input scans, ensuring robust performance without significant variance (<0.05 mm) in inlier RMSE.

**Figure 4:**
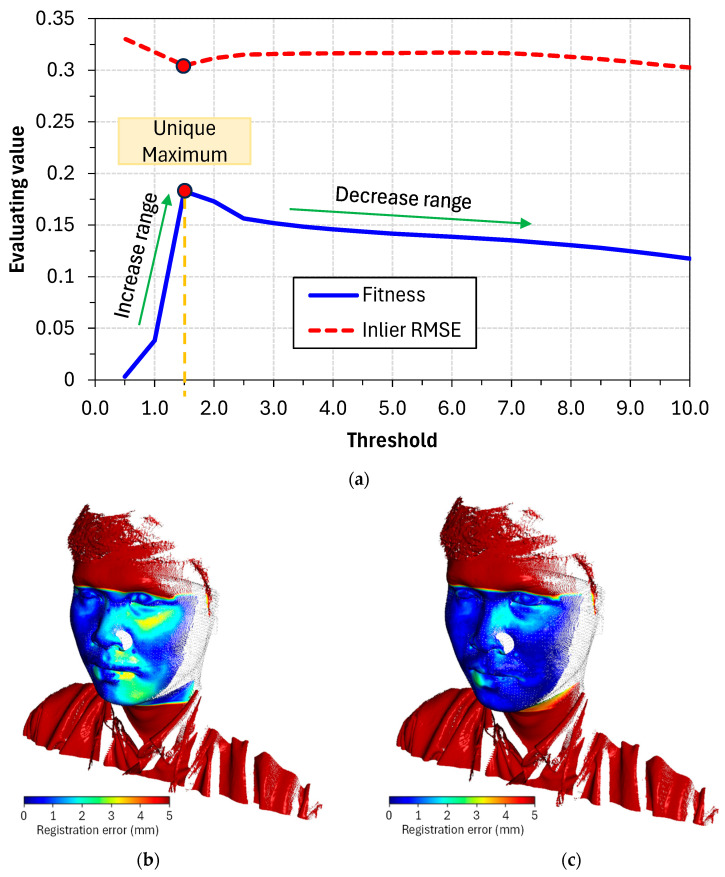
Demonstration of the similarity in the threshold—evaluation metric graph is found: (**a**) registration performance on thresholds; (**b**) registration error with threshold of 10; (**c**) registration error with threshold of 1.5.

Through graph analysis, as shown in Figure 4, the fitness response depended on the threshold, and an important property was identified. Following the decreasing trend of the threshold, fitness gradually increased because the objective function E(T) raised its criterion. However, when the threshold decreased beyond a certain point, fitness ceased to increase and instead dropped sharply to zero. In other words, within the threshold range of 0 to 10, a distinct maximum always exists.

Based on the identified features, an effective search strategy was proposed. The algorithm began calculations with threshold values starting at 10 and decreasing incrementally by one. During this process, the fitness value was monitored and expected to increase. The calculation continued until the fitness value began to decline, at which point the algorithm stopped and commenced a localized search. This localized search explored neighboring points with a finer step size of 0.5 to identify the configuration that yielded the highest fitness value, which was then considered the optimal threshold value. This search methodology, as shown in Algorithm 2, minimized the computation required for neighboring threshold values while maintaining the precision of a 0.5-step-size search. In addition, by incorporating downsampling, which reduced the number of points from the original dataset before commencing the calculations, this strategy significantly improved processing speed.
**Algorithm 2**: Pseudo-code for precise alignment based on the ICP-based method.Search for optimal threshold value1:** input**: 2:     Target point cloud P
3:     Source point cloud Q
4:${\;\;\;\;\;Thresh}_{0}=Max\_threshold\;(10)$5:     **repeat:**
6: 7: 8: 9: 10:        Find transformation matrix $T_{0,{Thresh\;}_{0}}$        ${Thresh}_{1}$ = ${Thresh}_{0}$ − 1
       Find transformation matrix $T_{1,{Thresh}_{1}}$
       Calculate ${Q_{0}}^{\prime }=T_{0,{Thresh\;}_{0}}\cdot Q$
       Calculate ${Q_{1}}^{\prime }=T_{1,{Thresh\;}_{1}}\cdot Q$11: 12: 13: 14:        Calculate ${fitness}_{0}$$\;(P,\;{Q_{0}}^{\prime })$
       Calculate inlier ${RMSE}_{0}(P,{Q_{0}}^{\prime })$
       Calculate ${fitness}_{1}$$\left(P,{Q_{1}}^{\prime }\right)$
       Calculate inlier ${RMSE}_{1}(P,{Q_{1}}^{\prime })$
15:${\;\;\;\;\;\;\;Thresh}_{0}$ = ${Thresh}_{0}-1$16:        **until** ${\;fitness}_{0}$ > ${fitness}_{1}$
17: 18: 19: 20: 21: 22: 23: 24: 25: 26: 27:${\;\;\;\;Thresh}_{0,{local}_{1}}$ = ${Thresh}_{0}+0.5$
${\;\;\;\;Thresh}_{0,{local}_{2}}$ = ${Thresh}_{0}-0.5$
    Find transformation matrix $T_{1,{Thresh}_{0,{local}_{1}}}$
    Find transformation matrix $T_{2,{Thresh}_{0,{local}_{2}}}$
    Calculate ${Q_{1}}^{\prime }\;=\;T_{1,{Thresh}_{0,{local}_{1}}}\cdot Q$
    Calculate ${Q_{2}}^{\prime }\;=\;T_{2,{Thresh}_{0,{local}_{2}}}\cdot Q$
    Calculate ${fitness}_{1}$$\;(P,\;{Q_{1}}^{\prime })$
    Calculate inlier ${RMSE}_{1}(P,{Q_{1}}^{\prime })$
    Calculate ${fitness}_{2}$$\;(P,\;{Q_{2}}^{\prime })$
    Calculate inlier ${RMSE}_{2}(P,{Q_{2}}^{\prime })$
28:**return**${\;Thresh}_{0,{local}_{i}}$ with ${fitness}_{i}=max({fitness}_{1},{fitness}_{2})$

## 3. Results

### 3.1. Demonstration of Registration Results

**Figure 5:**
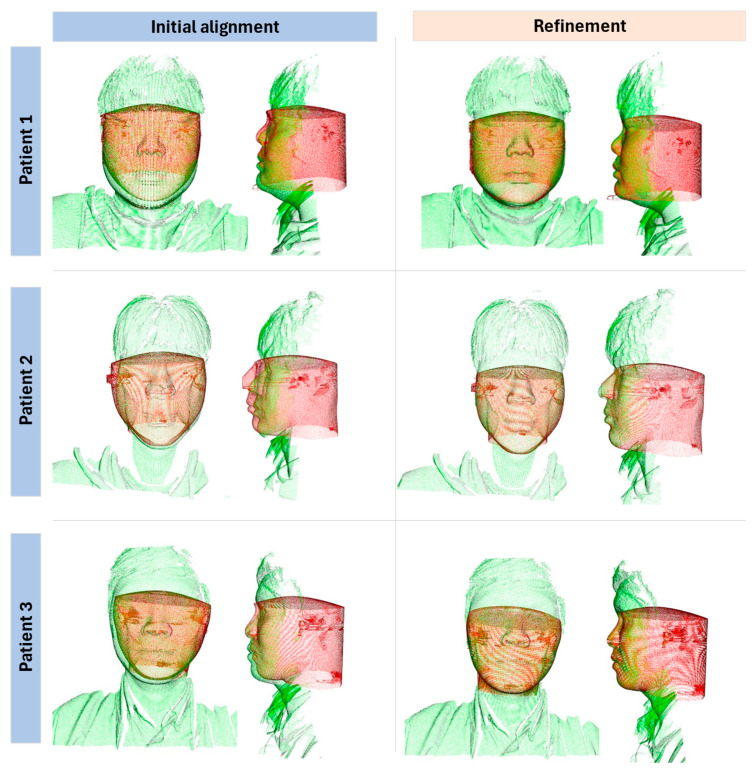
Automatic registration with initial alignment supported by face mesh detection and subsequent precise calibration with proposed ICP between CBCT (red) and face scan (green) data.

As shown in Figure 5, this section illustrates the efficiency of a two-step method consisting of initial alignment and subsequent refinement in data matching for patient imaging. The method employs the superposition of two distinct datasets, visualized in red and green, to demonstrate improvements in alignment accuracy at each step.

The first step, depicted in the left column for each patient, involves aligning the datasets based on preliminary estimates. This initial alignment successfully matched the two datasets but often lacked the precision required for clinical applications. The visible misalignments at this stage underscore the limited accuracy achievable with the initial alignment alone, highlighting the need for further refinement to meet higher precision standards.

In the second step, shown in the right column, the initial alignment is refined. By employing advanced processing techniques, including iterative methods and optimization algorithms, this step markedly improves alignment accuracy. The stark contrast between the images before and after refinement clearly illustrates the effectiveness of this method. For instance, in Patient 1’s images, the refinement process significantly enhanced the alignment, particularly around complex anatomical structures such as the head.

This two-step process efficiently addresses and corrects the inherent limitations of the initial alignment, ensuring that the final data alignment is both accurate and reliable for medical use. Improvements in dataset congruence from the initial to refined alignment underscore the necessity and utility of this method for enhancing diagnostic and therapeutic precision.

### 3.2. Registration Error Between Two Data Types

**Figure 6:**
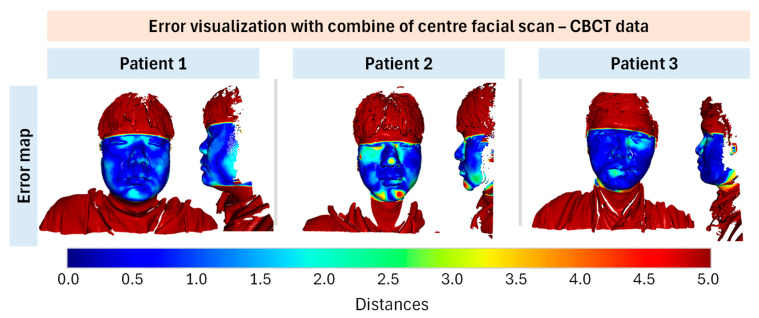
Error visualisation with centre facial scan point cloud using Jet colormap.

To enhance visualization, a method for directly viewing errors through color mapping is provided, as illustrated in Figure 6. Each point in the facial scan point cloud is assigned a color representing its distance from the corresponding point in the CBCT point cloud. The jet colormap was employed because of its high contrast and effectiveness in visualization, offering a smooth gradient from dark red (indicating a large distance) to dark blue (indicating a small distance).

An adjustment was made for points located far from the overlap region (facial area) that had significantly larger distances from their corresponding points. Because the colormap represents a fairly large range of values, the contrast for points with small distance differences can be reduced. Therefore, the upper limit was set to 5.0. The colormap represents the distance distribution between 0 and 5, where points near the blue end have errors close to zero and points near the red end have errors approaching 5.0. Points far outside the survey range were considered irrelevant and were displayed entirely in dark red.

In Figure 6, the datasets used to display the error for each patient are based on a previously determined optimal threshold. Using this optimal configuration, the evaluation values were determined, as listed in Table 1.

Upon observation, it was evident that most points on the patients’ faces were blue, indicating an error of approximately 0.5 mm. As previously mentioned, pairs of corresponding points with a distance less than 0.5 mm are considered inlier correspondences. Most blue points on the face were reflected in the fitness and inlier RMSE values. In this context, the fitness values indicated that 15–20% of the surface area overlapped, whereas the inlier RMSE provided valuable information by showing that, for valid pairs of points, the average error was approximately 0.3 mm (as shown in Table 1, where the inlier RMSE for all patients was around 0.3 mm). Beyond inlier RMSE, we also assessed complementary error metrics such as the 95th percentile error and Hausdorff distance, which more effectively capture local misalignments. These results are detailed in Supplementary Table S4. While the average inlier RMSE consistently remained ~0.3 mm across patients, local boundary regions exhibited Hausdorff distances exceeding 1 mm, particularly around the perioral and nasal areas. This suggests that the reliable portion of the point cloud data achieved an accuracy of approximately 0.3 mm.

An exception was observed in Figure 6, where Patient 2 exhibited several positions close to light red, indicating significant deviations (approximately 4.0 to 4.5 mm in error). These deviations were attributed to the lack of inlier correspondence at these positions, resulting in Patient 2’s fitness appearing lower than that of the other patients. However, it is important to note that this still represented the best achievable rigid registration based on the available input data. The observed large positional errors were likely due to deviations in the patient’s gestures caused by the lack of synchronization between the 3D facial scanning and CBCT data. This problem can be mitigated by using a synchronized dataset. By contrast, Patients 1 and 3 demonstrated that, with high-quality input data, the algorithm consistently identified the optimal alignment position, yielding smoother and more reliable error distributions across the face. This is a promising indicator of the potential for effective point cloud alignment and the capability to meet diagnostic requirements in clinical settings.

### 3.3. Effect of Face Scan Data with Respect to Multiple Capturing Directions

**Figure 7:**
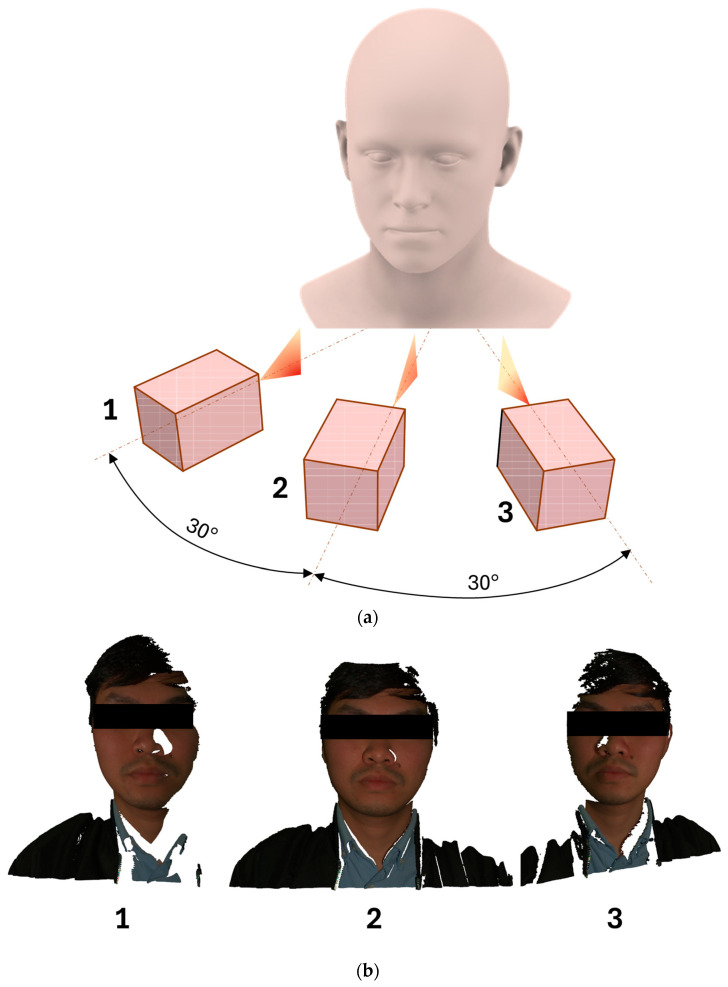
Face scan data from three capturing direction: (**a**) Capturing setup; (**b**) Example of three face scan data including left (1), centre (2), right (3).

In this study, the facial scan data were obtained using an RGB-Depth camera instead of a specialized full-head scanner. Consequently, the quality of the 3D data derived from the RGB-Depth camera was influenced by the capture direction. To assess the performance of the method across different capture orientations, three facial scans were obtained for each participant from various camera angles. These angles were spaced approximately 30° apart, as shown in Figure 7a. As depicted in Figure 7b, the facial scans captured from the left and right lateral directions exhibited omissions in several facial features. Notably, areas such as the opposite jaw, the alae of the nose, and the lateral aspects of the face were missing. In addition, portions of hair were not captured. These omissions occur because features closer to the camera can obstruct the view of those positioned behind them.

**Figure 8:**
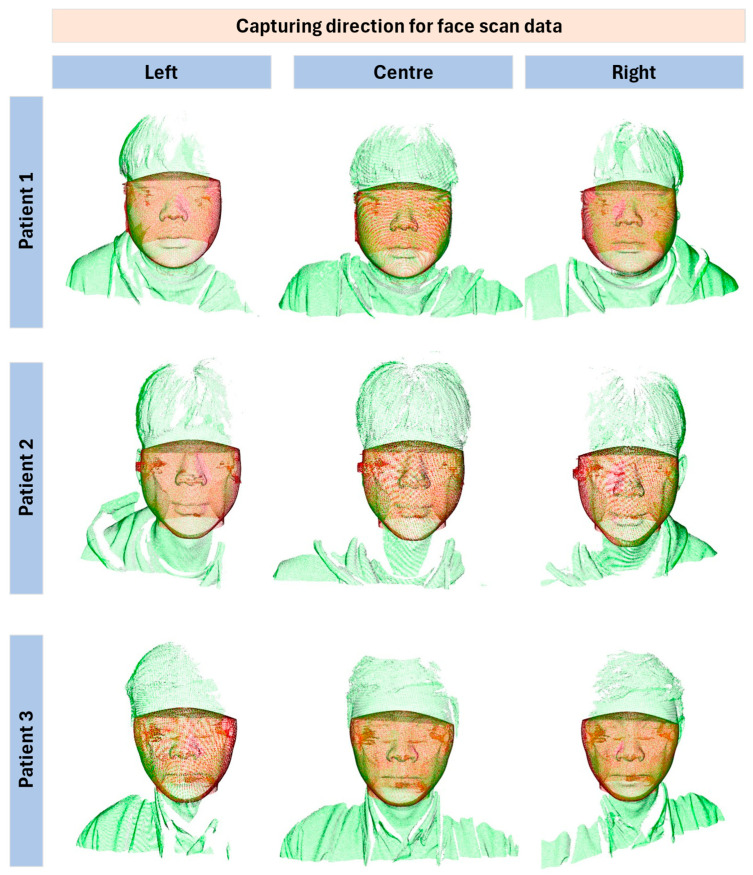
Registration results between CBCT and three face scan data captured from different camera direction (Red: CBCT, Green: Face scan data).

Figure 8 illustrates the outcomes achieved by applying the developed registration method to align the CBCT data with the facial scan data collected from multiple directions. Compared with the results from the central direction, it is evident that the proposed method produced consistent outcomes for facial scans obtained from the left and right directions. Across all the test cases, there were no instances of misalignment between the different capture directions.

Furthermore, despite the observable absence of certain features in the registration results, the left and right sides of the facial scan data were missing, with only the CBCT component remaining visible. Therefore, the quality of the registration results remained uniform. This consistency underscores the minimal impact of facial scan direction on the performance of the developed method.

## 4. Discussion

Based on the validation results of the developed method by registration for the face scan and CBCT data, the matching results demonstrated average inlier RMSE ~0.3 mm under our evaluation protocol. However, due to low overlap ratios (~15–20%), these values should not be over-interpreted as clinical accuracy. Future work will include fiducial-based target registration error, synchronized capture systems, and validation on larger cohorts. Limitations include small sample size, lack of anatomical ground truth, sensitivity to facial expressions and occlusions, and dependency on MediaPipe’s general-purpose training. Nevertheless, this feasibility study demonstrates the potential of face mesh–guided initialization for improving ICP-based multimodal registration.

While this feasibility study demonstrates promising results, the sample consisted of only three young adult male participants. This narrow cohort limits the generalizability of our findings across age ranges, sexes, and patients with diverse clinical presentations. In particular, facial hair, orthodontic appliances, surgical alterations, and other medical conditions may significantly affect surface quality and registration robustness. These factors must be systematically evaluated in future studies before clinical deployment.

Although inlier RMSE values remained consistently sub-millimeter, the fitness metric was relatively low (~0.15). This outcome mainly reflects the limited overlap between the full CBCT head volume and the partial facial surface scan, rather than failure of the registration algorithm. While local errors (RMSE, 95th percentile error, Hausdorff distance) confirmed alignment within sub-millimeter accuracy, the low fitness indicates that robustness must be carefully interpreted. Clinical feasibility will require further validation to assess how such overlap limitations influence treatment planning accuracy and reproducibility.

A prominent method involves the use of two-dimensional (2D) projection images to detect 3D facial landmarks, followed by fine registration using ICP. The primary advantage of this method is its high accuracy. By using 2D projections to detect landmarks, this method ensures precise alignment between the CBCT and facial scan data, which is crucial for applications such as 3D digital treatment planning and orthognathic surgery [18]. Additionally, the automation of this process reduces the need for manual intervention, thereby minimizing human error. However, this method is computationally intensive and requires significant processing power and time, particularly for high-resolution scans. Moreover, the accuracy of landmark detection can be affected by noise and artifacts in the 2D projection images, potentially leading to misregistration. This sensitivity to image quality necessitates high-quality input data, which may not always be available.

Deep learning techniques have been increasingly applied to the fusion of CBCT and intraoral scans to enhance the detail and accuracy of 3D dental models. A major advantage of using deep learning is its robustness. Deep learning models can handle various types of input data, thereby improving the overall robustness of the fusion process. Additionally, these models can significantly enhance the detail and accuracy of the resulting 3D models, which is beneficial for clinical applications [19]. However, deep learning models require large amounts of annotated training data, which can be difficult and expensive to obtain. This data-intensive requirement poses a significant challenge for the development and refinement of these models. Furthermore, the black-box nature of deep learning algorithms can render the decision-making process opaque. This lack of transparency can be problematic when verifying the accuracy and reliability of results, especially in clinical settings where precision is paramount.

CDFRegNet, an unsupervised network designed for CT-to-CBCT registration, addresses intensity differences and poor image quality in CBCT and achieves high registration accuracy. This method is advantageous because it does not require labeled training data, making it easier to implement and more flexible in various scenarios. It is particularly effective in handling typical issues associated with CBCT images, such as low contrast and noise [20]. However, similar to other complex models, CDFRegNet is computationally demanding. The training and execution of such networks require substantial computational resources that may not be readily available in all clinical settings. Additionally, although the method is unsupervised, it still relies on the quality of the input data and the architecture of the network, which means that suboptimal settings can lead to less accurate results.

The fusion of CT and 3D face scan data offers significant advantages, such as enhanced diagnostic accuracy by providing a comprehensive view of both bone and soft tissue structures and improved visualization, which aids in a better understanding of anatomical relationships. The integration of this method with IoT platforms facilitates real-time data sharing and remote diagnostics, which are beneficial for telemedicine [21]. In addition, it enhances patient comfort and safety by reducing the need for multiple imaging sessions, thereby minimizing radiation exposure. However, the process involves high computational complexity, requiring sophisticated algorithms and significant processing power to accurately align and merge the data.

A significant advantage of the proposed method over the feature point detection approach is its robustness to external regions, such as body parts below the neck, clothing, and other non-facial areas. Traditional methods often require manual or automated exclusion of these external regions or an expanded training dataset to accommodate these conditions in deep learning models. By contrast, the proposed method uses the automatic alignment of the input data based on face mesh detection, effectively mitigating the influence of these external regions. This approach ensures that registration performance remains unaffected by non-relevant areas, leading to more accurate and reliable outcomes.

In other research approaches, full-head data from both CBCT and face scans, encompassing both the face and back of the head, were required to extract features. However, this extensive data requirement is unnecessary for the proposed method. Our approach separately aligns the CBCT and face scan data based on the extracted face mesh. 3D data alignment is achieved gradually with continuous updates to the face mesh results and adjustments using a P-controller until convergence is reached. This method simplifies the process and enhances alignment accuracy without the need for full-head data.

However, this method presents two notable limitations. First, at the initial stage, the face mesh detection model must accurately recognize the patient’s face to initiate the alignment process. This necessitates an appropriate setup of the relative positioning between the 3D data and the virtual camera within the 3D environment to ensure reliable alignment. Second, the total alignment runtime is around 10 s, which is still suboptimal and warrants further reduction in future work. All experiments were performed on a workstation with an Intel^®^ Core™ i9-13900H CPU, 32 GB RAM, and an NVIDIA RTX 4070 Laptop GPU. The median runtime per case was 10.2 s, comprising approximately 2.5 s for pose initialization and 7.7 s for ICP refinement. A detailed breakdown of runtimes for each patient is provided in Supplementary Table S3.

In addition to methodological limitations, ethical and governance considerations are central to deploying automated CBCT–face fusion in clinical workflows. As emphasized by Kováč et al. [12], facial analysis systems must be designed with privacy-by-design principles and explicit consent management to mitigate risks of misuse or unauthorized re-identification. Our pipeline, though technically promising, must therefore be accompanied by institutional safeguards, audit trails, and transparent data governance protocols before integration into clinical decision-making.

## 5. Conclusions

This study presented a feasibility method for the fusion of CBCT and facial scan data, aiming to improve 3D facial alignment through face mesh–guided initialization and ICP refinement. The system automatically detects and aligns facial meshes from independently captured CBCT and face scan datasets. Our results demonstrated average inlier RMSE values of ~0.3 mm, with complementary error metrics supporting robustness in most facial regions. These findings should be interpreted as preliminary, given the small sample size, absence of fiducial-based ground truth, and dependence on pre-trained models. Future work will extend the evaluation to larger cohorts, ground-truth validation, and synchronized data capture.

The main contributions of this work can be summarized as follows:

1. By employing MediaPipe for coarse face mesh landmark detection, we achieved a reliable initial alignment that reduces the risk of local misalignment during refinement. This provided a more accurate starting point for ICP and improved robustness across varying capture conditions.
2. The use of the point-to-plane ICP algorithm, combined with an adaptive threshold search, ensured efficient and precise alignment with a processing time of ~10 s per case.
3. The proposed method demonstrates the feasibility of integrating CBCT and facial scan data for multimodal craniofacial analysis. Although global fitness values were relatively low due to the inherent limited overlap between CBCT (full head volume) and face scan data (frontal surface only), local error metrics consistently confirmed sub-millimeter alignment within the overlapping facial regions. This distinction highlights that low global fitness is a characteristic of the multimodal data rather than a failure of the registration process. While not yet clinically validated, the method provides a promising foundation for future applications in orthodontic planning, craniofacial surgery, and related clinical research.

## Figures and Tables

**Table 1:** Optimal threshold, fitness, and inlier RMSE for patients.

	Optimal Threshold	Fitness	Inlier RMSE
Patient 1	1.5	0.1826	0.3046
Patient 2	5.5	0.1419	0.2990
Patient 3	1.5	0.1847	0.2835

## Data Availability

Data is contained within the article or supplementary material.

## Supplementary Materials

The following supporting information can be downloaded at: https://www.mdpi.com/article/10.3390/bioengineering12090975/s1, Table S1: Participant demographics and scan instructions; Table S2: Sensitivity analysis of controller gain (p-gain) for pose initialization. Experiments were conducted on all three patients. Results are reported as mean ± SD across the three cases; Table S3: Runtime benchmarking for the three patients. All experiments were performed on a workstation equipped with Intel^®^ Core™ i9-13900H CPU, 32 GB RAM, and NVIDIA RTX 4070 Laptop GPU; Table S4: Registration error metrics for the three patients. Results are reported after ICP refinement with face mesh initialization.
