# Supplementary material for: AI-Assisted Fusion Technique for Orthodontic Diagnosis Between Cone-Beam Computed Tomography and Face Scan Data
Source: Bioengineering (Basel). 2025 Sep 14;12(9):975. doi: 10.3390/bioengineering12090975 (PMC12467118; doi:10.3390/bioengineering12090975)
Supplement: Supplementary file 1 [file bioengineering-12-00975-s001.zip › bioengineering-3842966-supplementary.pdf]

Supplementary Table S1. Participant demographics and scan instructions

Patient ID	Sex	Age (years)	Inclusion criteria	Exclusion criteria	Pre-scan instructions
Patient 1	Male	24	No prior facial surgery; no orthodontic appliances	Significant facial hair; medical conditions altering craniofacial anatomy	Neutral expression; no glasses; hair tied back
Patient 2	Male	28	No prior facial surgery; no orthodontic appliances	Significant facial hair; medical conditions altering craniofacial anatomy	Neutral expression; no glasses; hair tied back
Patient 3	Male	34	No prior facial surgery; no orthodontic appliances	Significant facial hair; medical conditions altering craniofacial anatomy	Neutral expression; no glasses; hair tied back

Supplementary Table S2. Sensitivity analysis of controller gain (p-gain) for pose initialization.
Experiments were conducted on all three patients. Results are reported as mean ± SD across the three cases.

p-gain value	Convergence success	Average iterations to converge	Residual rotation error (°)	Residual translation error (mm)
0.005	3/3 (100%)	62 ± 5	0.42 ± 0.08	0.36 ± 0.05
0.010	3/3 (100%)	48 ± 4	0.39 ± 0.06	0.34 ± 0.04
0.020	3/3 (100%)	44 ± 6	0.41 ± 0.07	0.35 ± 0.06
0.050	2/3 (67%)	38 ± 5	0.63 ± 0.11	0.49 ± 0.08

Interpretation:

- Gains below 0.005 led to very slow convergence.
- Gains above 0.02 occasionally caused instability and overshoot.
- **p = 0.01** provided stable convergence with the best trade-off between speed and accuracy.

Supplementary Table S3. Runtime benchmarking for the three patients.
All experiments were performed on a workstation equipped with Intel® Core™ i9-13900H CPU, 32 GB RAM, and NVIDIA RTX 4070 Laptop GPU.

Patient ID	Pose initialization (s)	ICP refinement (s)	Total runtime (s)
Patient 1	2.4	7.6	10.0
Patient 2	2.6	7.8	10.4
Patient 3	2.5	7.7	10.2
**Median [IQR]**	**2.5 [2.4–2.6]**	**7.7 [7.6–7.8]**	**10.2 [10.0–10.4]**

Supplementary Table S4. Registration error metrics for the three patients.
Results are reported after ICP refinement with face mesh initialization.

Patient ID	Inlier RMSE (mm)	95th percentile error (mm)	Hausdorff distance (mm)
Patient 1	0.3046	0.78	1.25
Patient 2	0.2990	0.95	1.68
Patient 3	0.2835	0.71	1.22

**Mean ± SD**	**0.2957 ± 0.011**	**0.81 ± 0.13**	**1.38 ± 0.25**

Interpretation:

- Inlier RMSE values are consistently ~0.3 mm.
- The 95th percentile errors (<1.0 mm) demonstrate robustness for most facial regions.
- Hausdorff distances are higher, reflecting local misalignments at boundary regions (e.g., perioral, nasal).
